# Third-party punishment-like behavior in a rat model

**DOI:** 10.1038/s41598-024-71748-x

**Published:** 2024-09-27

**Authors:** Kanta Mikami, Yuka Kigami, Tomomi Doi, Mohammed E. Choudhury, Yuki Nishikawa, Rio Takahashi, Yasuyo Wada, Honoka Kakine, Mayuu Kawase, Nanae Hiyama, Hajime Yano, Naoki Abe, Tasuku Nishihara, Junya Tanaka

**Affiliations:** 1 Department of Molecular and Cellular Physiology, Ehime University Graduate School of Medicine, Shitsukawa, Toon, Ehime 791-0295 Japan; 2 Department of Anesthesia and Perioperative Medicine, Ehime University Graduate School of Medicine, Shitsukawa, Toon, Ehime 791-0295 Japan

**Keywords:** Behavior, Morality, Wistar, Prefrontal cortex, Triage, Social behaviour, Animal behaviour, Prefrontal cortex

## Abstract

Third-party punishment (TPP) is an altruistic behavior or sense willing to punish transgressors as a third party not directly involved in the transgression. TPP is observed worldwide, regardless of tradition and culture, and is essential for morality in human society. Moreover, even preverbal infants display TPP-like judgement, suggesting that TPP is evolutionarily conserved and innate. Thus, it is possible that non-human animals display TPP-like behavior, although TPP has been said to be human-specific. We investigated whether or not male mature Wistar rats displayed TPP-like behaviors when they witnessed deadly aggression by an unknown aggressive mouse toward another unknown victim mouse. Normally reared rats did not display TPP-like behaviors, but rats reared with extensive affectionate handling by human caretakers as beloved pets contacted the unknown aggressive mice in a gentle manner leading to reduced aggression toward the unknown victim mice, even when the aggressive mice fought back. Furthermore, the handled rats touched unknown rat pups that were drowning in water and anesthesia-induced comatose rats more frequently than control rats. These findings suggest a possibility that TPP is not in fact human-specific and innate but rather an acquired behavior that flourishes in affectionate circumstances.

## Introduction

When witnessing violent acts, people usually hope that the perpetrator will be punished and the victim rescued or compensated^1^. Similar feelings arise even when the perpetrator and victim are both strangers, and the observer is a third party. This type of punishment is called third-party punishment (TPP), wherein individuals punish a wrongdoer or transgressor even when they themselves are unaffected third parties. TPP is at the core of social norms observed worldwide, spanning different traditions and cultures and playing an important role in sustaining human society and fostering cooperation between humans^2–5^. Not only adults but also toddlers and even infants before they can speak long for TPP for violators of fairness norms^6–8^. A recent study demonstrated that preverbal eight-month-old infants held by their mothers made attacking motions when viewing a cartoon face bullying another cartoon face, indicating TPP^9^. Furthermore, six-month-olds have the ability to judge whether an action is good or bad simply by viewing the interactions of simple-shaped cartoon figures^9–11^. Preverbal infants have also been shown to make moral judgments through observations that the infants prefer characters (animal hand puppets) that play prosocial behavior positively and that negatively behave toward antisocial individuals^12^. The same authors found this to be true even for three-month-olds^12,13^. These studies suggest that a sense of morality or justice leading to helping the weak and interfering with aggressive behavior may be innate rather than fostered after birth.

The notion that TPP is innate as well as the fact that TPP is commonly observed in human societies worldwide suggest that it may be an evolutionarily developed behavior or sense. If this is the case, TPP-like behaviors, even in undifferentiated forms, should be present in non-human animals as well. However, TPP is said to be specific to humans and has not been observed even in chimpanzees, which are our closest living relatives^14^. Chimpanzees perform second-party punishments, in which the victim directly punishes the conspecific wrongdoer. However, when one chimpanzee watches another stealing a victim’s food, it does not try to punish the wrongdoer, indicating that chimpanzees do not punish others as third parties. However, some papers argue that primates show TPP-like behaviors, although these behaviors may not be TPP itself^15–17^. Similarly, dogs, fish, and other animals have been shown to display third-party evaluations or TPP-like interventions, although these actions are unlikely to be true TPP^6,18–20^.

Considering the various reports describing prosocial behaviors and/or undifferentiated TPP-like interventions in non-human animals, we assessed whether or not mature male Wistar rats enacted TPP by creating a TPP test (TPPT). In the TPPT, rats were allowed to witness aggressive bite attacks by Jc1:ICR (ICR) mice toward C57BL/6JJc1 (BL6) mice. Aggression by ICR mice often causes severe injury or even death in BL6 mice, and this is often used as a social defeat stress model to study depression, mental stress or sleep disorders^21^. Normally reared rats did not show any particular intervening behaviors; however, rats reared with extensive affectionate handling (EAH) on a daily basis for weeks by caretakers, as if they were beloved pets, displayed TPP-like behaviors that reduced aggression in ICR mice in a rather gentle manner. We also investigated the possibility that the TPP-like behaviors of EAH rats were grounded in dispositions valuing lives using the newly created drowning test (DT) and triage test (TT), in which the EAH rats’ behaviors toward unknown conspecifics subjected to life-threating situations were observed. EAH rats exhibited touching behaviors toward the struggling and comatose conspecifics. We also found that EAH induced changes in gene expression in the medial prefrontal cortex (mPFC), which presumably correlated with increased neuronal activity.

## Results

### Rearing with extensive affectionate handling (EAH)

**Fig. 1:**
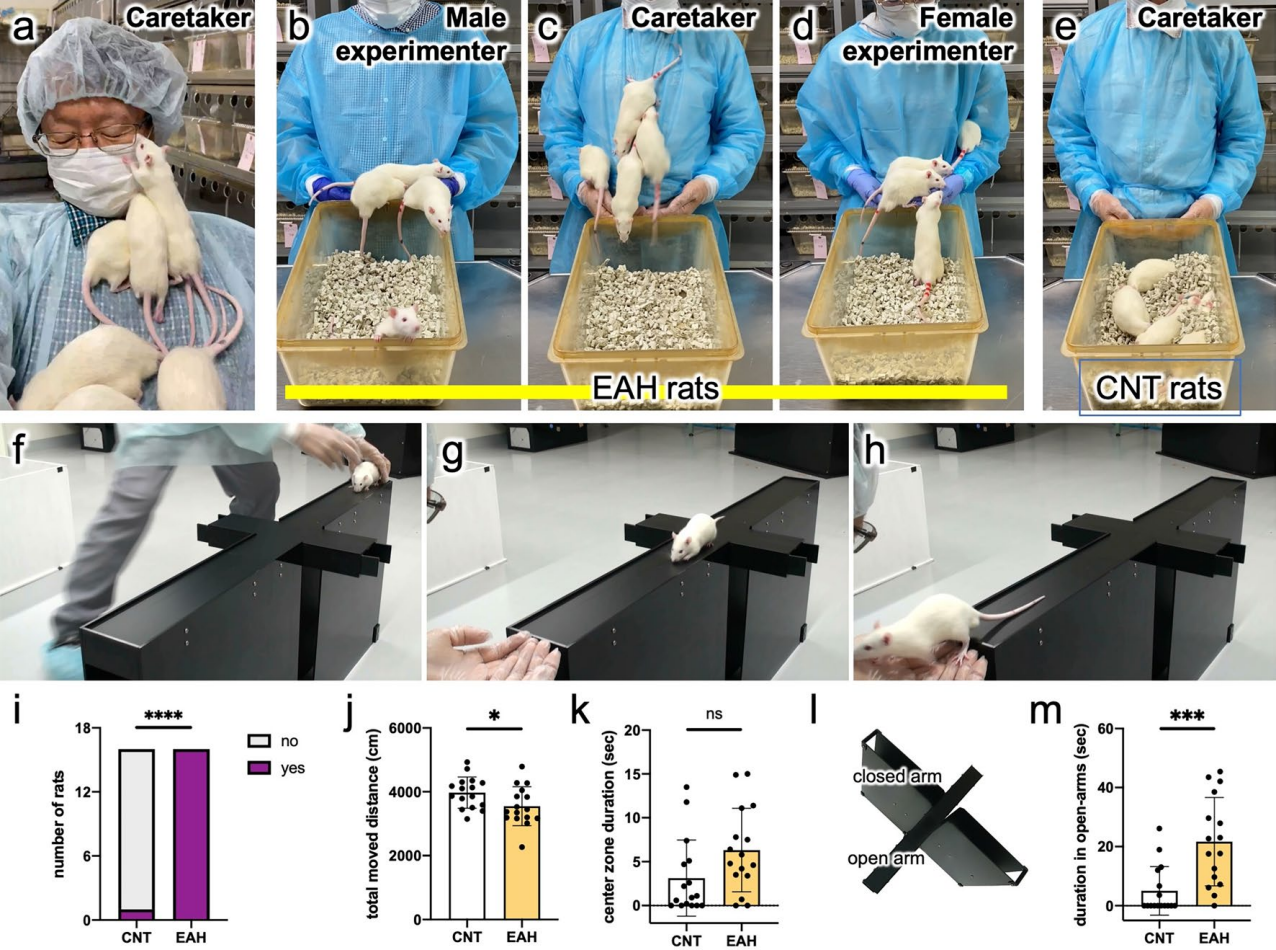
Extensive affectionate handling (EAH). (**a**) In EAH, rats were allowed to move freely across the caretaker’s body. (**b**–**d**) EAH rats showed familiarity to humans with their palms outstretched in front of a cage while simultaneously seeming to distinguish the caretaker (**c**) from unknown experimenters (**b**: male; **d**: female). (**e**) CNT rats did not approach the caretaker. (**f**–**i**) The attachment test (AtT): the caretaker gently placed an EAH rat on one end of the open arm of the elevated plus maze (EPM) (**f**), moved to the other end, and held out both hands, calling “Come!” or “Rick (the name given to all of the rats)” (**g**); if all 4 of the rat's legs were on the hands within 90 s, the session was considered to have been successfully completed (**h**). All 16 EAH rats but only 1 CNT rat reached the hands of the caretaker; ‘yes’ and ‘no’ denote succeeded and failed in the AtT, respectively (**i**). χ^2^ test and Fisher’s exact test. (**j**, **k**) The open-field test (OFT): total moved distance (**j**) and the duration staying in the center zone of OF arena (**k**). Unpaired two-tailed *t*-test. (**l**, **m**) An EPM apparatus (**l**). EAH rats stayed longer on the open arms than CNT rats (**m**). Unpaired two-tailed *t*-test. *n* = 16. *, ***, **** indicate statistical significance at *p* < 0.05, 0.001, and 0.0001, respectively.

It is widely known that handling rats or mice causes changes in their behaviors, even when handling is rather brief^22–24^. In this study, EAH was performed for male Wistar rats by a male caretaker in his 60 s who cared for and played with the rats as if they were his beloved pets for 15 min a day, 6 or 7 times per week after weaning on postnatal day (PND) 21. All rats were named “Rick,” which was the caretaker’s dog name. Within a few days of starting EAH, the rats began to gather around the caretaker’s hands when he approached them (Fig. S1a). They moved freely on the body or around the neck of the caretaker (Fig. 1a). The rats sometimes communicated with rats in different cages and of different ages for their enjoyment (Fig. S1b). The EAH rats showed strong familiarity with the caretaker, but also gathered around the palms of unknown male and female experimenters (Fig. 1b–d; Suppl. Fig. S2a). In contrast, normally reared control (CNT) rats, which were members of the litters of the EAH rats, did not approach the caretaker (Fig. 1e; Suppl. Fig. S2b).

To evaluate the attachment of the rats to the caretaker, an attachment test (AtT) was performed, wherein a rat was placed on one end of an open arm of an elevated plus maze (EPM; Fig. 1f–h; Supplementary Video 1 [SV1]) apparatus set in a brightly lit laboratory that the rats had never been in before. The rats were called by a caretaker from the other end; among the 16 rats in each group only one CNT and 16 EAH rats got on the caretaker’s hands within 90 s (Fig. 1i). Behavioral characteristics of the rats were investigated using the open-field test (OFT) and EPM. CNT rats showed slightly increased mobile activity and similar anxiety levels compared to EAH rats, as revealed by the OFT (Fig. 1j, k). However, the EPM results suggested that EAH rats were less anxious than CNT rats (Fig. 1l, m).

### The third-party punishment test (TPPT)

**Fig. 2:**
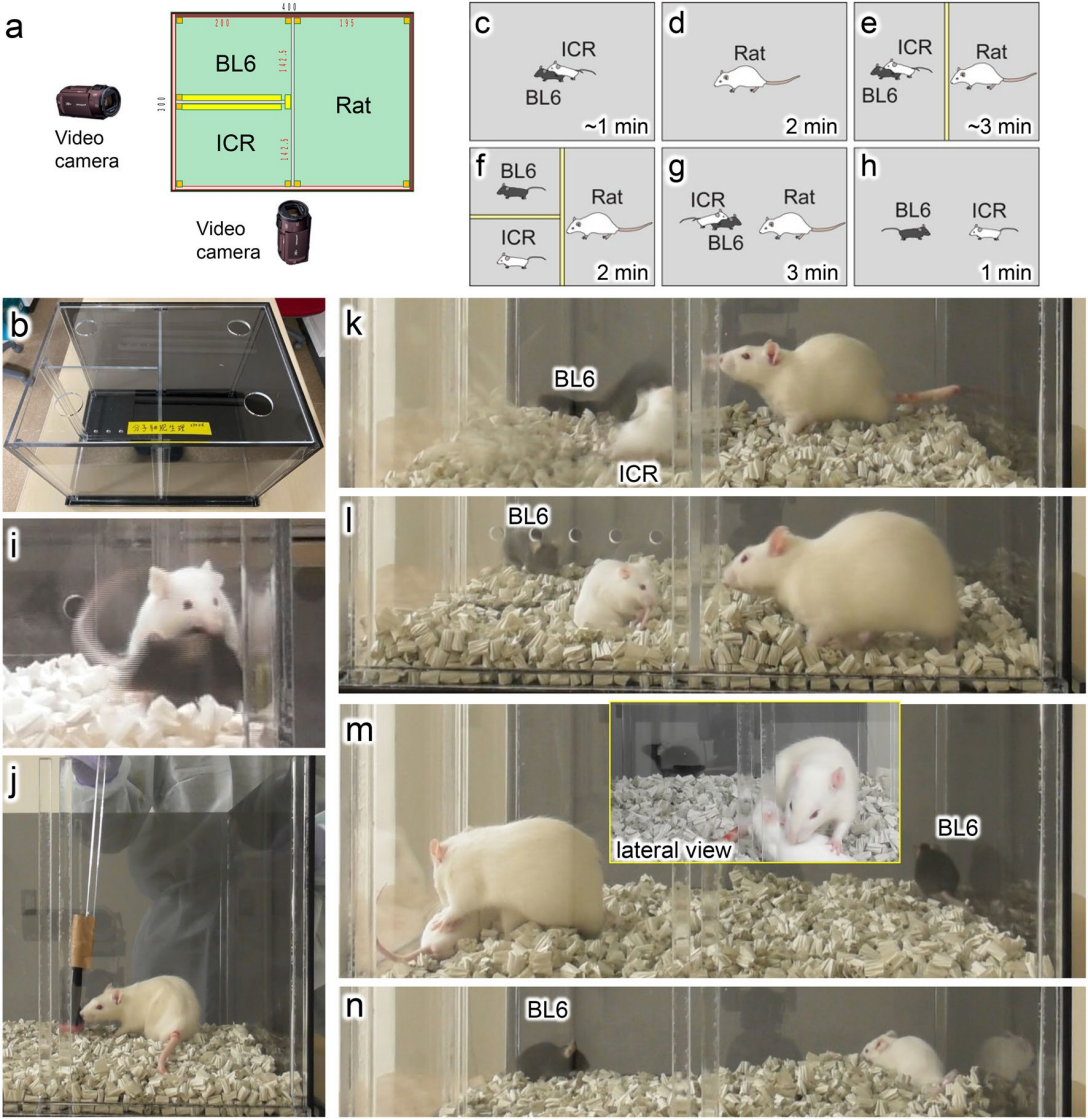
Apparatus and procedures of third-party punishment test (TPPT). (**a**, **b**) The TPPT apparatus. Values are in millimeters (**a**). Two video cameras were set in the front and the side of the apparatus. (**c**–**n**) The observation procedure of TPPT in the case of an aggressive mice pair. All of the steps were videorecorded. (**c**, **i**) Confirming that the ICR (white) and BL6 (black) mice pairs showed aggressive behaviors in the apparatus. The time (min) shown in figure, (**c**–**h**) denotes observation duration. (**d**, **j**) An actor rat was released for habituation to the apparatus and the brush attached to the acrylic rod. (**e**, **k**) A transparent partition was inserted to divide the mouse and rat compartments, and the mice and rats were placed in each compartment. The rat was allowed to watch the aggression by the ICR mouse toward the BL6 mouse for 3 min. (**f**, **l**) Inserting a partition between the two mouse compartments to stop the aggressive behavior by the ICR mouse and waiting for 2 min for the animals’ excitement to subside. (**g**, **m**) All partitions were removed, and the behaviors of the animals were observed for 3 min. (**h**, **n**) After the rat was removed, the behaviors of the mice were observed for 1 min.

**Fig. 3:**
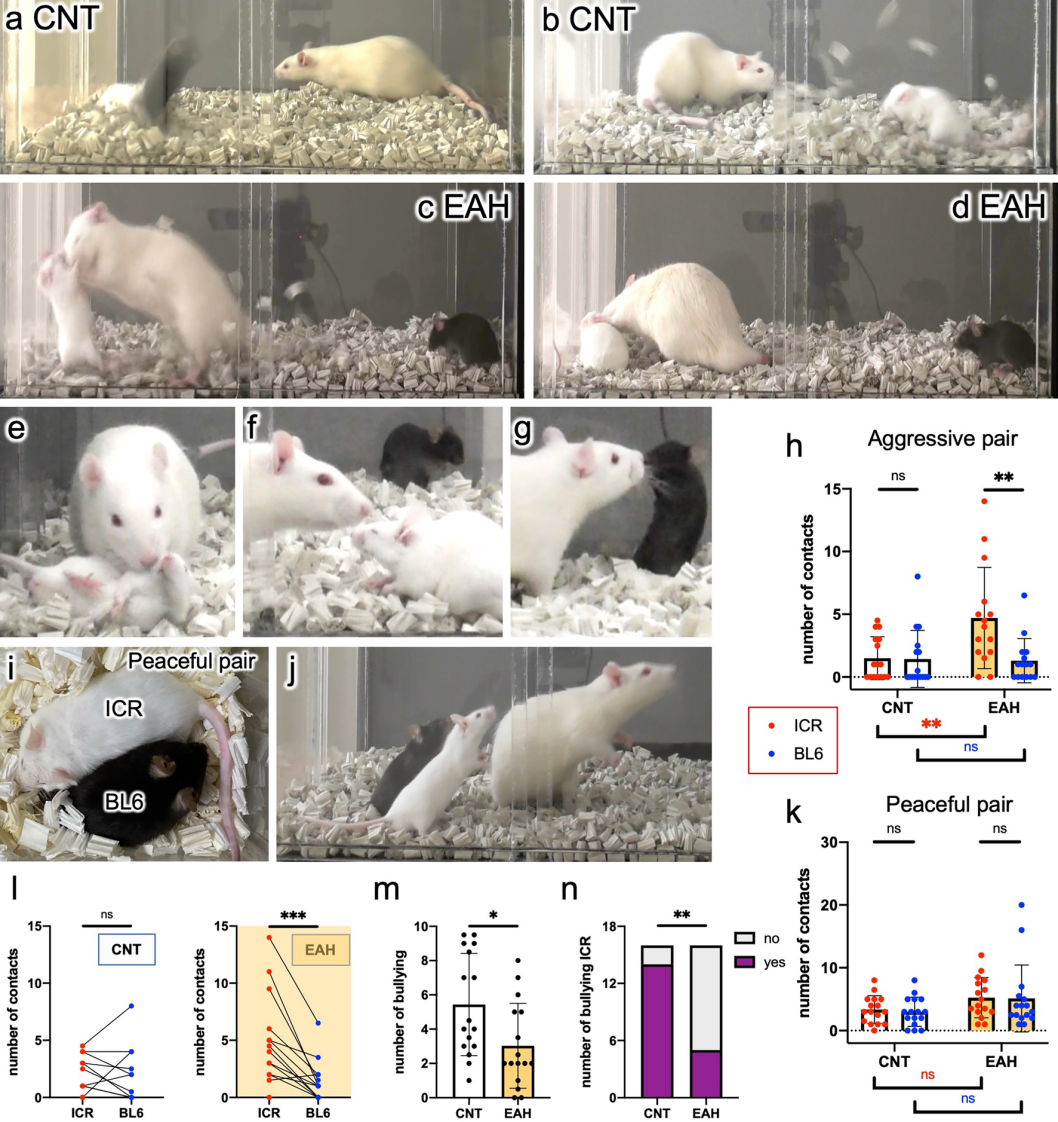
Third-party punishment test (TPPT) results. (**a**, **b**) Behaviors of two representative CNT rats in the TPPT. The CNT rats avoid approaching the mice. The two video captures show instants when ICR mice attacked BL6 mice. (**c**, **d**) Behaviors of an EAH rat. The rat suffered a retaliatory strike by an ICR mouse (**c**), at which point the rat nonviolently contacted the aggressive mouse (**d**). (**e**–**g**) Another EAH rat pressed down a violent ICR mouse with its forepaws but did not bite the mouse (**e**). The ICR mouse decreased its aggressive behaviors toward the BL6 mouse in the presence of the EAH rat (**f**). The rat approached the BL6 mouse but did not frequently touch it (**g**). (**h**) EAH rats showed more frequent contact with the aggressive ICR mice than with the BL6 mice compared to CNT rats. (**i**, **j**) When presented with a peaceful pair of mice, there were no fights at all in the absence (**i**) or presence (**j**) of an EAH rat. (**k**) When presented with peaceful pairs, the number of contacts did not significantly differ between the CNT and EAH rats. (**l**) Most EAH rats made contact more frequently with aggressive ICR mice than with BL6 mice; however, there was no significant difference regarding frequency of contact made by CNT rats. (**m**) The ICR mice reduced the number of attacks made in the presence of the EAH rats. (**n**) The number of ICR mice that attacked the BL6 mice within 1 min after rat removal; ‘yes’ and ‘no’ denote attacked and not-attacked in the TPPT, respectively. CNT, n = 16; EAH, n = 16; only the EAH group data in **h**, n = 15. (**h**, **k**) A two-way ANOVA and Tukey’s post hoc test; paired (**l**) or unpaired (**m**) two-tailed *t*-test; (**n**), χ^2^ and Fisher’s exact test.

**Fig. 4:**
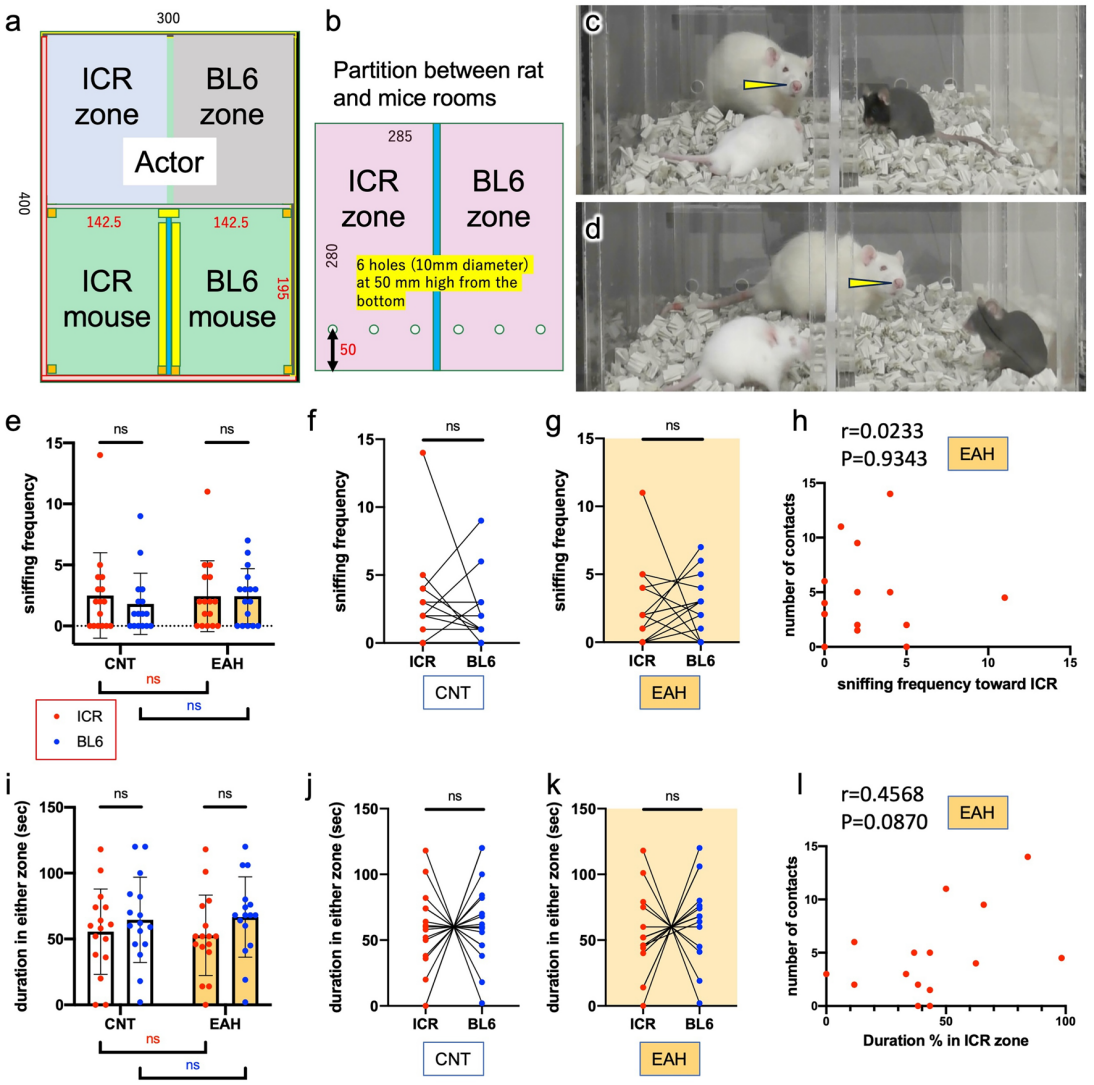
The actors’ attitudes toward the mice after witnessing the aggression in TPPT. (**a**) Definition of ICR and BL6 zones in the rat compartment in the TPPT apparatus; the former was adjacent to the ICR compartment, and the latter the BL6 compartment. Values are in millimeters. The ICR and BL6 zones were switched for each experiment. (**b**) The partition between the rat and mice compartments with holes. (**c**, **d**) The actor rats could sniff the mice through the holes drilled the partition set between the rat and the mice compartments by inserting their nose tips. Arrowheads denote the sniffing nose tip through the holes. (**e**) Sniffing frequency by actors toward ICR or BL6 mice was counted. (**f**, **g**) Paired t analysis on the sniffing frequency by EAH (**f**) and CNT (**g**) rats toward attacker ICR or victim BL6 mice. (**h**) Correlation between sniffing frequency and number of contacts with ICR mice by EAH rats. (**i**) Duration stayed in either ICR or BL6 zones. (**j**, **k**) Analysis on the duration by EAH (**f**) and CNT (**g**) rats in the ICR or BL6 zones with paired *t*-test. (**l**) n = 16; except for the data in **h** and **i** (n = 15). (**e**, **i**) A two-way ANOVA and Tukey’s post hoc test; (**f**, **g**, **j**, **k**) paired two-tailed *t*-test; (**h**, **l**) Pearson correlation coefficient and two-tailed Student's *t*-distribution.

Actor rats’ behaviors when witnessing aggression by unknown mice were evaluated using the TPPT (Figs. 2, 3 and 4). The TPPT was performed according to the procedures shown in Fig. 2, using an apparatus with three compartments that could be partitioned by transparent acrylic removable boards (Fig. 2a, b). The actor rat was allowed to watch the aggression of an ICR mouse toward a BL6 mouse through a partition (Fig. 2e, k). After inserting all partitions to divide the animals individually and waiting for 2 min for their excitement to subside (Fig. 2f, l), all partitions were removed to allow the three animals to make contact with each other (Fig. 2g, m). The behavior of the animals was evaluated by counting the number of times the rats made contact with either the ICR mouse or the BL6 mouse and the number of attacks made by the ICR mouse. Furthermore, after the rat was removed, we assessed whether or not the ICR mouse attacked within 1 min (Fig. 2h, n).

CNT rats did not approach either the aggressive ICR mice or victim BL6 mice (Fig. 3a, b, h; SV2). In contrast, EAH rats frequently interacted with ICR mice and stopped them attacking by holding the ICR mice down with their forepaws in a rather gentle manner (Fig. 3c–h; Fig. S3; SV3, SV4), even though the EAH rats often suffered retaliatory strikes by the ICR mice (Fig. 3c). The EAH rat shown in Fig. 3c and d contacted the ICR mouse 11 times and the BL6 mouse only once during the observed 3 min. On average, EAH rats made contact with BL6 mice less frequently than ICR mice (Fig. 3g–l), and there were no significant differences in the number of contacts with BL6 mice between CNT and EAH rats (Fig. 3h). The individual rat’s behavior toward an ICR mouse and an BL6 mouse was analyzed with paired t-test (Fig. 3l). EAH rats but not CNT rats contacted ICR mice more frequently rather than BL6 mice. Of note, EAH rats were associated with a reduced number of attacks by ICR mice during their stay (Fig. 3m) as well as after their removal (Fig. 3n). One EAH rat that lunged at the ICR mouse promptly after removal of the partitions frequently had to be stopped with a brush; this result was excluded from the aggression pair data (Fig. S4) because of the associated difficulty counting the number of contacts.

The day after the TPPT was performed with aggression pairs, the tests was performed again with peaceful pairs not showing any aggressive behaviors (Fig. 3i) using the same CNT and EAH rats. With the peaceful pairs, both the EAH rats and mice remained calm (Fig. 3j), and the number of contacts with ICR and BL6 mice by both CNT and EAH rats (Fig. 3k) did not differ significantly.

To investigate the reason why the EAH rats approached the ICR mice and reduced their aggression, the actors’ behaviors were observed and quantified for 2 min while the animals were individually partitioned after the actors’ watching the aggression through partition (Fig. 4). ICR and BL6 zones were defined in the rat compartment; the former was adjacent to the ICR’s compartment, the latter BL6’s (Fig. 4a, b). Duration staying of their heads in either zone was measured. Three holes with 10 mm diameter were drilled in the partition between the rat and mice compartments. The actor rats could sniff at the mice through the holes by putting their noses into the holes (Fig. 4c, d). The sniffing frequency at either ICR or BL6 mice was counted (Fig. 4e–g). On the average, both CNT and EAH rats did not display specific interest in either ICR or BL6 mice. The sniffing frequency was not correlated with the number of contacts by EAH rats with the aggressive ICR mice (Fig. 4h). There were no significant differences in the staying duration of the rats in either zone (Fig. 4i–k). The staying duration of EAH rats in the ICR zone was weakly correlated with the number of their contacts with the attacker ICR mice, although there was no statistical significance (Fig. 4l). The results shown in Figs. 3 and 4 suggest that EAH rats showed no particular interest in the attacker ICR mice when there were no aggressive behaviors.

### The drowning test (DT)

**Fig. 5:**
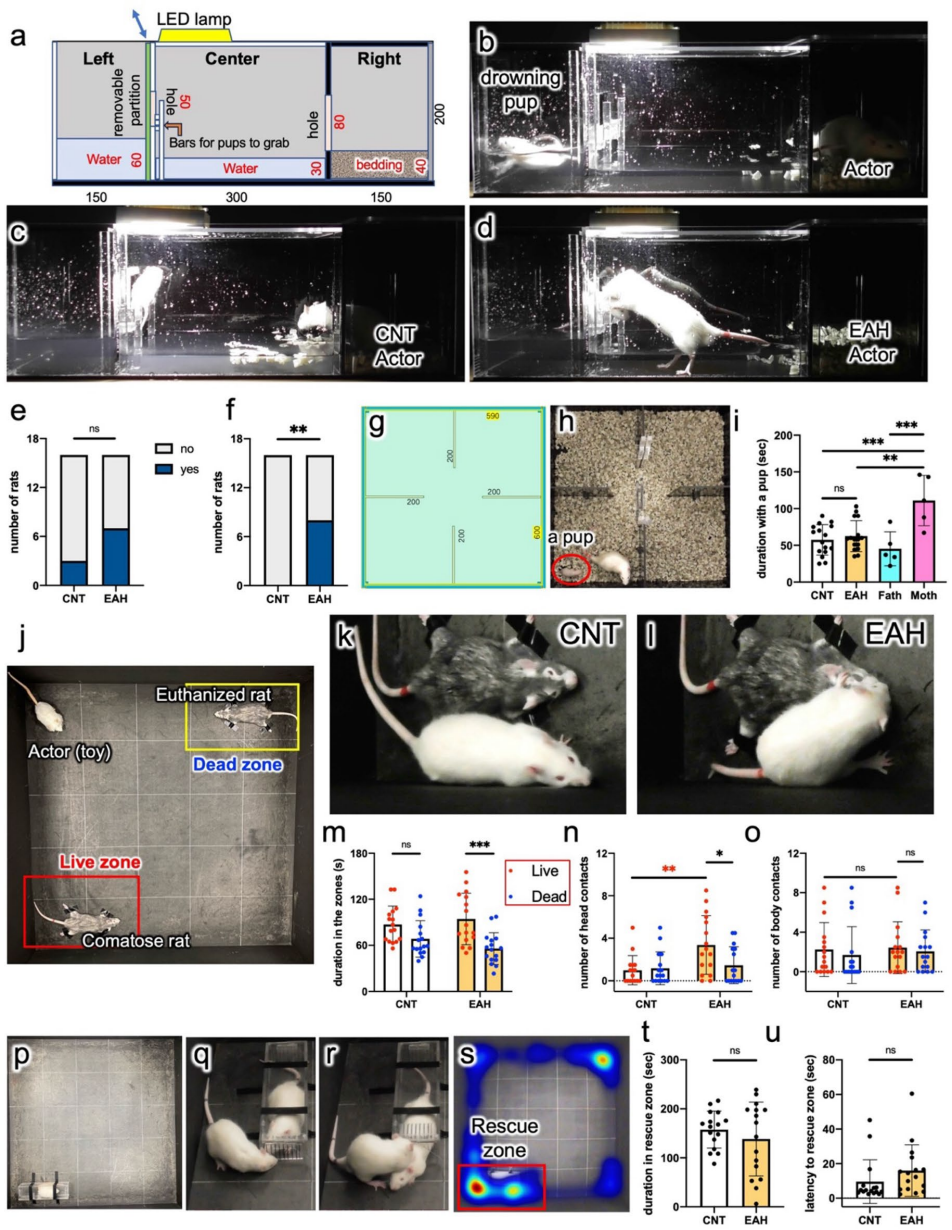
The drowning test (DT), 4-room test (4RT), triage test (TT), and rescuing-restrained-rat test (RarT). (**a**–**d**) DT: the apparatus (**a**). Values are in millimeters. A drowning PND16 pup and an actor rat (**b**). Response of a CNT rat (**c**) and an EAH rat (**d**; see SV5). The number of rats that touched the partition between the left and center chambers during habituation; ‘yes’ and ‘no’ denote touched and not-touched the partition, respectively (**e**) and those that made contact with the drowning pups; ‘yes’ and ‘no’ denote contacted and not-contacted the pups, respectively (**f**). (**g**–**i**) 4RT: the 4RT box (**g**). Colocalization of an actor and a PND11 pup (**h**). Duration (sec) of the colocalization (**i**). Mother (Moth) rats but not Father (Fath) rats stayed with their pups for longer periods than CNT and EAH rats. (**j**–**o**) Triage test (TT): setting (**j**). Video captures showing behaviors of a CNT rat (**k**) and an EAH rat (**l**) in the live zone. Duration (sec) in the live and dead zones (**m**). Number of contacts with the head (**n**) and the body (**o**). EAH rats stayed longer in the live zone than in the dead zone and made contact with the head of comatose rats more frequently than with the head of euthanized rats. (**p**–**u**) RarT: setting (**p**). A restrained rat was placed in a live zone of the TT. A rescued rat (**q**, **r**; Fig. S8). Setting of the rescue zone corresponding to the live zone in the TT and a representative heatmap showing movements of an EAH rat over 5 min (**s**). Duration (sec) in the rescue zone (**t**) and time until the first entry (sec) into the rescue zone (**u**). *n* = 16. (**e**, **f**) χ^2^ test and Fisher’s exact test; (**i**) a one-way ANOVA and Tukey’s multiple comparison test; (**m**–**o**) a two-way ANOVA and Sidak’s multiple comparison test; (**t**, **u**), unpaired two-tailed *t*-test. *, **, *** indicate statistical significance at *p* < 0.05, 0.01, and 0.001, respectively.

To gain insight into why the EAH rats stopped the aggression they witnessed in the TPPT, the newly created DT was performed. In the DT, the touching behavior of actor rats toward unknown drowning PND16 pups in water was observed using an acrylic box with three chambers with passages for the actor rats (Fig. 5a–d). The left and center chambers were then filled with water. An LED light was placed above the center chamber with an illuminance of 11,100 lx on the floor. The right chamber was separated by a black opaque acrylic panel and covered with bedding made of paper. Thus, if the actor rat wished to touch or to rescue the drowning pup, it would need to surmount the obstacles of water and bright illumination.

First, the actor was placed in the right chamber for 3 min of habituation. Although no significant difference was noted, a greater number of EAH rats entered the center chamber than did CNT rats during habituation (Fig. 5e). A transparent partition was set between the left and center chambers, and a PND16 pup unknown to the actor was placed in the left chamber. The drowning pup moved violently in an effort to escape (Fig. S5; SV5), and the actor observed this situation for 1 min. Following removal of the partition, half of the EAH rats touched the drowning pup within 30 s; however, none of the CNT rats approached it (Fig. 5c, d, f; Fig. S5; SV5).

To examine whether or not the touching behaviors during the DT emerged out of concern for the lives of the pups or merely as a result of the curiosity of each EAH rats, we established a 4-room test (4RT) using a square acrylic box, the interior of which was divided into four compartments using opaque black acrylic panels (Fig. 5g, h). The movements of the actor rat and a PND11 pup were video recorded for 5 min, and their colocalization duration in the same compartment was measured. For comparison, we also measured the duration of colocalization of parent rats with their pups as well (Fig. 5i). Mother rats but not Father rats stayed with pups for longer periods than CNT and EAH rats. The 4RT showed that EAH rats did not demonstrate any particular interest in the pups.

### The triage test (TT)

To gain further insight into whether or not EAH rats would try to rescue unknown rats in life-threatening situations, the TT was created, wherein an actor rat was released into an open-field arena where two male Wistar littermate rats unknown to the actors had been placed, one of which had been euthanized while the other was only under deep anesthesia (Fig. 5j). Both live and dead zones were established around the anesthesia-induced comatose rat and the euthanized rat, respectively, and the time spent in each zone and the number of contacts made with the euthanized/comatose rats’ heads were measured. In the TT, EAH rats significantly stayed longer in the live zone than in the dead zone (Figs. 5k, l, m, S6, S7). EAH rats made contact with the heads of comatose rats more frequently than did CNT rats, with the heads of comatose rats more frequently than those of euthanized rats, and with the heads of comatose rats more frequently than their bodies (Figs. 5n, o; S7). These findings suggest that EAH—but not CNT rats— prioritized the comatose rats rather than the euthanized rats while distinguishing the comatose rats from the euthanized ones.

### Evaluating rats’ empathy

To investigate whether or not the EAH rats’ prosocial behaviors are correlated with empathy, the behaviors of CNT and EAH rats toward an unknown restrained male rat of the same age were observed using a test called the rescuing-restrained-rat test (RarT; Fig. 5p–u; Fig. S8). This is similar to a previously established experiment to evaluate empathy ^25^, except the rectangular container for restraining an unknown rat was of simpler construction, with front and rear doors that could be easily opened by pushing from the outside. However, if the restrained rat attempted to escape from the inside, the door would not open, so cooperation between the two rats was required. The container was placed at the periphery of an open-field arena (Fig. 5p). None of the CNT and EAH rats rescued the restrained rats within 5 min. Notably, one CNT rat successfully rescued the restrained rat within 6 min (Fig. 5q, r; Fig. S8). Such rescue behaviors in the RarT may be based on rats' empathy, taking previous notions^25,26^. To evaluate the attitude of the actor rats leading to the rescue behavior, we measured the duration spent in the zone around the rectangular box (rescue zone; Fig. 5s) and the latency to the first entry into the zone by the actor rats. However, these data did not differ significantly between the CNT and EAH groups (Fig. 5t, u), suggesting that EAH may not have strengthened empathy that was evaluated by the present RarT.

### Effects of different rearing conditions on rats’ behaviors

**Fig. 6:**
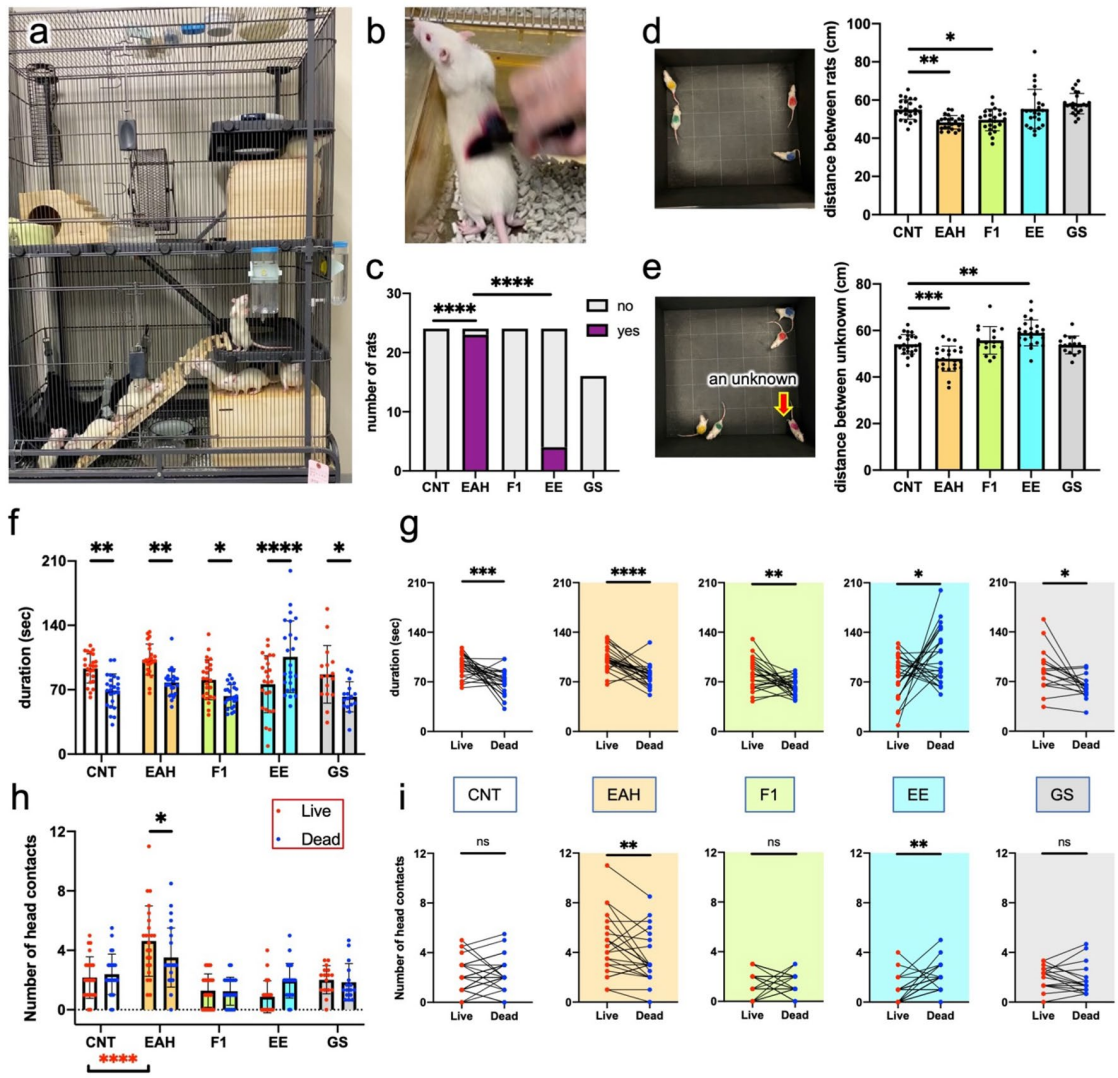
Various rearing methods and behavioral changes in the AtT, GOFT, and TT. (**a**) Group rearing of 12 rats in an enriched environment (EE) prepared in a complex housing. (**b**) Rearing with gentle stroking (GS): rats gently stroked on the back with a cosmetic brush. (**c**) Attachment test (AtT). Rats reared with 5 different methods were subjected to AtT; ‘yes’ and ‘no’ denote succeeded and failed in the AtT, respectively. F1: offspring of EAH rat parents. (**d**, **e**) Grouped OFT (GOFT): four cage mates were released to the OF, and the mean distance between the rats was measured for 5 min (**d**). (**e**) GOFT with an unknown rat: mean distance between the cage mate and the unknown rat. (**f**) Duration spent in the live or dead zone in the triage test (TT). (**g**) The comparison of the duration spent in the live and dead zones by individual rats. (**h**) Number of contacts with the heads of comatose and euthanized rats in TT. (**i**) The comparison of the number of contacts with the heads of the comatose and euthanized rats by individual rats. *n* = 24, except for GS group (*n* = 16). (**c**) χ^2^ test and Fisher’s exact test; (**d**, **e**) a one-way ANOVA and Tukey’s multiple comparison test; (**f**, **h**) a two-way ANOVA and Sidak’s multiple comparison test; (**g**, **i**) paired two-tailed *t*-test. *, **, ***, **** indicate statistical significance at *p* < 0.05, 0.01, 0.001, and 0.0001, respectively.

To gain more insight into the effects of the rearing environment on prosocial behaviors, rats reared in five different conditions were subjected to behavioral tests, including the TT. The five conditions were as follows: (1) CNT, (2) EAH, (3) rearing in group of 12 rats in an enriched environment prepared in complex housing (EE; Fig. 6a), (4) normal rearing of offspring (F1) of EAH rats, and (5) rearing with chronic gentle stroking of the back (GS; Fig. 6b). The CNT and EAH rats were different from those used in previous studies. The EAH rats were reared by two caretakers (a male in his 60 s and female in her 20 s) for 30 min to 1 h a day, 6 or 7 times per week. In the AtT, of the 24 rats, no CNT rats, 23 EAH rats, no F1 rats, and 4 EE rats climbed onto the hands of the male caretaker, and none of the 16 GS rats exhibited this behavior (Fig. 6c). With findings that were somewhat different from those of the previous results shown in Fig. 1, the OFT showed that EAH rats were more active and less anxious than the CNT rats. This difference may be caused by the different EAH durations by two caretakers. On the other hand, EE rats were less active and more anxious than CNT rats (Fig. S9). Rats in the same cage underwent the grouped OFT (GOFT) (Fig. 6d), and the mean distance between the two rats was shortest in the EAH group. When an unknown male Wistar rat of the same age was added to the four cage mate rats, the mean distance between this unknown rat and the individual cage mate rat was the shortest in the EAH group (Fig. 6e).

The GOFT results suggested that EAH improved the social nature of rats. According to the TT, CNT, EAH, F1, and GS rats remained in the live zone longer than in the dead zone, whereas only EE rats remained longer in the dead zone than in the live zone (Fig. 6f, g). EAH rats made contact with the comatose rats’ heads more frequently than rats in the other groups (Fig. 6h, i). Furthermore, EAH rats more frequently made contact with the heads of comatose rats than with euthanized rats, while EE rats contacted those of euthanized rats more frequently than those of comatose rats.

### EAH-induced changes in gene expression in the medial prefrontal cortex (mPFC)

**Fig. 7:**
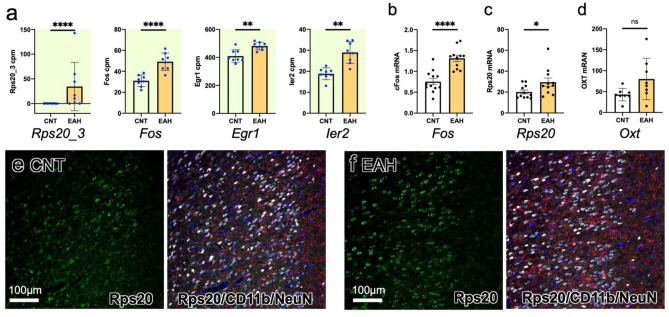
EAH-induced changes in gene expression in the mPFC. (**a**) Significant results from comparison of the gene expression in the medial prefrontal cortex (mPFC) between CNT and EAH rats were obtained by RNAseq; *Rps20_3*, *Fos*, *Egr1*, and *Ier2*. (**b**, **c**) Increased *Rps20* (**b**) and *Fos* (**c**) expression in EAH brains as revealed by qPCR. (**d**) Gene expression encoding oxytocin (*Oxt*) in the hypothalamus did not significantly change. (**e**, **f**) Rps20 protein expression was found in neurons (**e**, CNT; **f**, EAH brains) (green; Rps20, white; a neuronal marker NeuN, red; a microglia marker CD11b). Unpaired two-tailed *t*-test. *, **, ***, **** indicate statistical significance at *p* < 0.05, 0.01, 0.001, and 0.0001, respectively.

After the behavioral tests, the CNT and EAH rats were subjected to molecular biological analyses. Previous reports have indicated that the mPFC^5,27–29^ and hypothalamus (HPT) containing paraventricular and supraoptic nuclei secreting oxytocin (OXT)^30,31^ are involved in prosocial behaviors. We dissected the mPFC and HPT from CNT and EAH rat brains the day after the final behavioral test (TPPT using peaceful pairs) was done. Before sacrifice, EAH rats were handled by the caretaker as usual. Total RNA was extracted from the tissues. RNA sequencing (RNA-seq) data on the gene expression in the mPFC showed that only four genes were significantly different between CNT and EAH rats: *ribosomal protein s20_3* (*Rps20_3*), *Fos*, *Early Growth Response 1* (*Egr1*), and *Immediate Early Response 2* (*Ier2*) (Fig. 7a). These four genes were expressed at higher levels in EAH rats than in CNT rats. The increased expression of *Fos* and *Rps20* in the mPFC of EAH rats was confirmed by quantitative reverse transcription polymerase chain reaction (qPCR; Fig. 7b, c). Expression of the gene encoding oxytocin [*oxytocin/neurophysin I prepropeptide* (*Oxt*)], in HPT did not significantly differ between CNT and EAH rats (Fig. 7d). Immunohistochemical staining showed that Rps20 immunoreactivity was localized in NeuN^+^ neurons (Fig. 7e, f).

## Discussion

TPP, which refers to punishment of aggressors by uninvolved bystanders, has been said to be unique to humans^5^. Chimpanzees do show second-party punishment (where the victim is also the actor) but not TPP^14^. However, our TPPT results showed that rats displayed TPP-like behaviors toward aggressive ICR mice attacking victim BL6 mice but only when the rats had been reared with EAH for weeks after weaning. It might be probable that EAH rats contacted the attacker mice merely because of their interest in the mice but not with the aim to interfere aggressive behaviors. Yet, taken the observation that the actor rats did not show more interest in the attackers than the victims after the actors witnessed the aggressive behaviors, it is unlikely that the actors’ contacts with the attackers were generated just by the interest in the attackers.

The experimental paradigm of the present TPPT is principally similar to that involving showing cartoons to eight-month-old infants, an approach employed in a previous report^9^. In that study, infants held by their mothers watched an animated sequence in which a square face attacked another square face of a different color. After watching the wrongdoing on a monitor, infants were able to punish the wrongdoer by gazing at the face, which led to a stone being dropped on the aggressor face. In the TPPT, the rats first watched the attack by ICR mice on BL6 mice through a transparent partition, and then the rats were allowed to make contact with the mice directly. Compared to the test for infants using animation, the TPPT may be a more difficult task, as ICR mice often retaliated against the EAH rats. Thus, EAH rats intervened in the aggression by ICR mice while also overcoming the possibility of injury incurred from counterattacks. Except for one EAH rat that aggressively attacked the ICR mice without any direct provocation toward the rat, all other EAH rats never violently (such as biting) stopped the attacks by ICR mice, even when the mice made counterattacks. The EAH rats did not show any particular interest in ICR mice when encountering peaceful pairs one day after watching the aggressive ICR mice. These observations support the notion that most EAH rats tried to stop the aggression of ICR mice toward BL6 mice as a third party. Considering the nonviolent stopping of aggressive ICR mice, the EAH rats might have not aimed to punish ICR mice but rather to help BL6 mice. In fact, they gently approached the victim BL6 mice while interfering the attack. This observation of the rats’ behavior may be similar in part to a previous report demonstrating that preschoolers prioritize the prosocial behavior of returning stolen items to their victims over the punitive behavior toward moral offenders^32^.

To gain insight into the motives behind the behaviors of EAH rats in the TPPT, we next performed the DT. Half of the EAH rats approached and touched the unknown drowning pups despite the obstacles of bright light and water. The touching behavior seems to not have been the result of casual interest in the pups, as the rats did not show particular interest in the pup in the 4RT. The RarT did not demonstrate any significant difference between EAH and CNT rats, suggesting that empathy may not be correlated with the results of the TPPT. However, the RarT is a newly established test to compare empathy of CNT and EAH rats in a much shorter period than the reported method^25^, because many behavioral tests were loaded to the animals. Therefore, it may still be necessary to investigate whether the RarT is suitable to evaluate rats’ empathy. In the TT, EAH rats more frequently touched comatose rats than euthanized rats and did so the most frequently among rats reared under various conditions. Although the intention of EAH rats touching the comatose rats was unclear, EAH rats prioritized the comatose rats rather than the euthanized rats, while distinguishing life from death. The improved sociality of EAH rats may be correlated with their TPP-like behaviors, based on the GOFT results.

Riedl et al.^14^ showed that chimpanzees do not punish the conspecific as a third party when the victim’s food is stolen, even when the victim is a relative to the actor chimpanzees. However, the actors retaliate against conspecifics who have stolen the actors’ food, as a second party^14^. Notably, stealing food does not put the victim’s life at risk. Conversely, studies on preverbal infants utilize animations that involve physically harming others^9^. In the present TPPT, BL6 mice were violently attacked by ICR mice, often leading to severe injury or death if left attended. For the EAH rats, stopping the aggression of ICR mice provided no material gain, actually incurring a cost of potentially being harmed by retaliatory strikes. When combining the present results with those of previous reports, TTP behaviors by non-human animals or preverbal infants might mainly be directed toward others in life-threatening situations. It would be worth examining the behaviors of non-human animals, such as dogs or chimpanzees, who have been loved by humans, toward deadly violence between smaller animals that may otherwise pose no threat to the actors.

The ventromedial PFC (vmPFC) may play a role in TPP behavior as revealed by human imaging studies^27,28^. The mPFC, including the vmPFC, has been implicated in various prosocial behaviors^33,34^ as well as neurodevelopmental disorders^29,35^. Therefore, we compared the gene expression between the EAH and CNT rat mPFC using RNA-seq. However, only four genes (*Rps20_3*, *Fos*, *Egr1*, and *Ier2*) were expressed at higher levels in the mPFC of EAH rats than in that of CNT rats. *Rps20* encodes a protein that constructs the small ribosomal subunit^36^. Therefore, the increased expression of Rps20 suggests increased protein synthesis in the mPFC neurons of EAH rats. Increased expression of the immediate early genes *Fos*, *Egr1*, and *Ier2* may be indicative of elevated neuronal activities^35,37,38^ in the EAH rat mPFC, which might have led to the TPP-like behaviors seen in these animals. We did not detect a significant increase in *Oxt* gene expression in the hypothalamus, suggesting that Oxt-mediated empathy^31^ was not responsible for the rat behavior in the TPPT, in accordance with the results of the RarT.

Although no comprehensive changes in gene expression were found, the biological study indicated that rearing with EAH caused apparent changes in brain activity. The caretakers engaged in EAH said that they truly loved the rats and enjoyed the time spent with them. In the AtT, the rats appeared to climb into the hands of the caretakers happily in response to being called. Thus, the affectionate rearing environment appear to have promoted behaviors in TPPT overcoming counterattacks by ICR mice as well as the behavioral changes in DT and TT.

In conclusion, the present results suggest that the rats’ TPP-like behaviors may be acquired through exposure to affectionate environments and are not actually innate. The TPP behaviors of preverbal infants may be due to the love received from their parents or other caregivers. In fact, an electrophysiological study showed that the preference of children 12–24 months old for prosocial characters was influenced by their parents’ individual dispositions regarding justice and fairness, altering their children’s temporal neural dynamics^39^.

## Materials and methods

### Animals

Breeding of rats and mice and all animal experiments conformed to the Guidelines of the Ethics Committee for Animal Experimentation of Ehime University in Matsuyama, Japan. All animal experiments were also conducted according to ARRIVE guidelines. Euthanasia of animals was done according to the ​American Veterinary Medical Association​ Guidelines for the Euthanasia of Animals (2020)​. The animal experiments were approved by the Ethics Committee for Animal Experimentation of Ehime University (Approval No.: 05U43-2; 05U47-2; 05U46-1, 2). All routine care measures, such as bedding changing, feeding, and breeding, were performed very carefully only by the male caretaker (in his 60 s) to avoid variation in the routine rearing of animals.

#### Rats

Wistar rats were purchased from CLEA Japan, Inc. (Tokyo, Japan). Nine-week-old male and female Wistar rats were allowed to cohabitate, gestate, and give birth. Only males were used in our experiments. Pups subjected to our behavioral experiments were kept with their parents until postnatal day (PND) 21. After separated from their parents on PND21, pups were reared in one of the following five rearing methods: CNT, EAH, F1, rearing in a large cage group with enriched environments (EE), and gentle stroking (GS) group. Except for the EE group, the pups were reared in a standard plastic cage (40 × 25 × 14 cm), with four pups per cage, and behavioral experiments were conducted when they were 8–10 weeks old. Food and water were provided ad libitum, and room lighting (240 lx near the cage) was switched on from 7:00 to 19:00, and off at other times. The temperature of the room was 25 ± 1 °C, and the humidity was approximately 55%.

#### Five rearing methods for rats

1) CNT: Even-numbered male siblings of the same litter were divided into two groups and assigned to the CNT and EAH groups. The CNT rats were reared under normal rearing conditions.

2) EAH: The EAH group was consisted of a litter the same as that of the CNT group. The floor bed material made of paper was changed thrice a week, and pellet food was spread on the floor daily so that the rats could eat the food with forepaws. The rats were not dangled by their tails, even when changing cages. The EAH actions were as follows: frequently talking using words such as “cute,” “smart,” “come here,” and “see you tomorrow” as much as possible to the rats; holding and petting them, allowing them to freely play on the caretakers’ chest and belly, and allowing rats to play with other EAH rats of different ages (Fig. S1). The EAH was started at PND21 and done between 16:30–19:00 daily until the end of the behavioral testing.

3) F1: Four male and four female Wistar rats, born to different parents, were reared from PND21 to PND63 using the EAH method. At 8–9 weeks old, they were mated to prepare four pairs and allowed to give birth. The male pups (24 pups in total) delivered were separated from their parents on PND21, and they were then reared in the same manner as the CNT group.

4) GS: Starting at PND21, rats’ backs were gently stroked with a cosmetic brush for 10 min daily according to a previously described method^40^. Otherwise, the rats were reared in the same manner as the CNT group.

5) EE: Twelve pups were reared in a large, tall cage (95 × 55 × 115 cm) equipped with a running wheel, four stages, three wooden pens, a wooden ladder, plastic slopes, a climbing steel tube, three trays with food pellets, and two trays filled with water from PND21 until PND70 as has been described^29^.

#### Breeding of unknown rats for behavioral experiments

The male Wistar rats subjected to TT, RarT, and GOFT were born to parents with whom the actor rats had never been in contact, and were subsequently separated from their parents at PND23. Thereafter, they (four animals per cage) were reared in the same manner as the CNT group and used for the behavioral tests at 8–9 weeks of age. For 4RT and DT, male pups born to parents that were never in contact with the actor rats, were used at PND11 and PND16, respectively.

#### Mice

ICR and BL6 mice pairs were purchased from CLEA Japan, Inc. To prepare aggressive pairs of male ICR and BL6 mice, an acute social defeat stress model was employed according to the method utilizing the resident-intruder paradigm as describe elsewhere^21^. BL6 mice were individually and randomly placed into the ICR cage. We then observed whether the ICR attacked the BL6 or not. Repeating this temporary cohabitation, which triggers ICR attacks, we identified 16 aggressive mice pairs. Prior to TPPT, the ICR and BL6 were placed in the TPPT apparatus to confirm that the ICR attacked the BL6 within 1 min. When the ICR gave excessively violent attacks on BL6, the two animals were separated using a cosmetic brush that was attached to an acrylic rod (Figs. 2 and S3). Furthermore, two ICR-BL6 pairs that did not exhibit any aggressive behaviors were designated as peaceful pairs. While lighting on/off, feeding, water supply, and other conditions were the same as for the rats, the rooms for the rats and mice differed. Routine care was carefully performed only by the male caretaker. The ICR and BL6 mice were used for TPPT until they were 13 and 12 weeks old, respectively.

### Behavioral tests

Except for AtT, behavioral experiments were not performed by the caretakers but by other experimenters (male and female undergraduates in their 20 s; Figs. 1, S2) who had not touched the animals until the behavioral tests. No handling was used to calm the rats or mice before any of the behavioral experiments, except for the EAH rats. Behavioral experiments were conducted from 17:00 to 21:00. For the behavioral tests shown in Figs. 1, 2, 3, 4 and 5, the tests were performed in the following order in two weeks: AtT, OFT, EPM, TPPT, DT, 4RT, TT, and RarT. For the tests shown in Fig. 6, they were conducted in the following order: AtT, OFT, TT, and GOFT.

#### Attachment test (AtT)

The male caretaker placed a rat at one end of an open arm of the EPM (100 × 10 × 50 cm) in a brightly lit laboratory (1220 lx on the open arm) that the rats had never been in before. Then, the caretaker immediately moved to the other end of the arm and said “Come” or “Rick” to the rats. Normally, when rats were placed at the end of the arm, the rats were held by the body, but in the case of the CNT and other groups that did not like to be touched, the rats were held by the tail. If the rats’ four legs got on the hands of the caretaker within 90 s, the test was judged as successful (Fig. 1f–h, SV1). If the rat did not come to the hand by 90 s, we terminated the test. Some of the tests were recorded using a video camera (HC-W590MS; Panasonic, Osaka, Japan).

#### Open-field test (OFT)

A 5 min OFT was conducted using a square box (100 × 100 cm) with 50 cm-high walls. We used a video-tracking system (Ethovision XT 14; Noldus Info. Tech., Wageningen, The Netherlands) to monitor the rats’ movement and measure the following parameters: total moved distance and latency to the first entry into the center zone (60 × 60 cm)^41^. The illumination at the center of the OF arena was 54 lx.

#### Elevated plus maze (EPM)

The open and closed arms of the EPM apparatus made of black opaque acrylic boards were used in this experiment^35^. Closed arms were walled in with 50 cm-high black opaque acrylic plates. The test was started by placing the rats at the center of the apparatus. The video-tracking system was used to record rats’ movements for 5 min and measure the duration of staying in the open arms. The illuminance was 53, 75, and 6.6 lx at the center of the apparatus, end of the open arms, and end of the closed arms, respectively.

#### Grouped open-field test (GOFT)

Four rats in the same cage or five rats in the group with four rats plus one unknown male rat of the same age were simultaneously released into the abovementioned OF (1 m square; Fig. 6d, e), and their behavior was recorded using the video-tracking system for 5 min. Red, blue, green, yellow, and pink were painted on the rats' backs for recognition by the video-tracking system. The averaged distance between each rat from the four-rat group or between the unknown rat and the four rats in the case of the five-rat experiment was measured.

#### Triage test (TT)

Two littermates (male Wistar rats at 8–9 weeks old) that were unknown to the test rats were prepared per eight test rats; one was euthanized by carbon dioxide inhalation, and the other was injected intraperitoneally with 0.3 ml per 100 g body weight of a mixture of medetomidine hydrochloride (0.75 ml; Kyoritsu Pharmaceutical, Tokyo, Japan), midazolam (2 ml; Astellas, Osaka, Japan), butorphanol tartrate (2.5 ml; Meiji Seika Pharma, Tokyo, Japan), and saline solution (7.25 ml) to be comatose. The whole bodies of the two rats were painted black to prevent misidentification by the video-tracking system. These two rats were placed in the corner symmetrical to the OF, and their limbs were fixed to the bottom surface with a black vinyl tape. The illumination was the same as that in the OFT. A 40 × 25 cm area around the comatose and euthanized rats was designated as the live zone and dead zone, respectively (Fig. 5j). The test rats were released from the other corner, and their behavior was recorded using the video-tracking system; additionally, two video cameras were simultaneously used to record the test rats' behavior around the live and dead zones. The video-tracking system was used to measure the total distance traveled and the duration of stay in the two zones. Two undergraduate students and a laboratory secretary, who were all not involved in the experiment and were unaware of the experimental conditions, observed the video recordings and measured the number of times the test rat contacted the head (above the shoulder) or body (below the shoulder) of the comatose or euthanized rat by the nose, mouth, or forepaw. From the measurements of three persons, two data with close values were extracted and averaged to obtain the results.

#### Drowning test (DT)

An acrylic box (60 × 20 × 20 cm) with three chambers was made (Fig. 5a). The left and right chambers were 15 cm wide, and the center chamber was 30 cm wide. The rear and right sides are separated by translucent gray acrylic panels, through which an experimenter observed the animal behavior. Moreover, the center and right chambers were separated by an opaque black acrylic panel with 8 cm square hole, where the rat could pass. We also partitioned the center and left chambers by using a removable transparent acrylic panel with 5 × 7 cm rectangular hole. The left chamber was filled with water at 6 cm deep, and the center chamber at 3 cm deep. The water temperature was 23–25 °C. The right chamber was covered with a dry, unused floor mat made of paper. A 15W LED lamp was placed above the center chamber. The illuminance of the box was 11,100, 6,660, 15.3, and 1,350 lx at the center chamber under the light, the left chamber, the right chamber in shadow, and the right chamber in light near the square hole, respectively. A video camera was set in front of the apparatus to record the animals' behavior. In the experiment, rats were first placed in the right chamber and allowed to freely move in the apparatus for 3 min for habituation. After inserting a partition between the left and the center chambers, we placed an unknown rat pup of PND16 into the water in the left chamber and left it there for 1 min. Then, the partition was removed, and the behaviors were recorded until the pup escaped into the right chamber. Two persons who did not know the experimental conditions watched the video and examined the following two points: whether the rats left the right chamber and touched the boundary between the center and left chambers during the habituation, and whether the test rats touched the pups within 30 s after the partition removal. DT was terminated after a maximum of 3 min to prevent hypothermia of the rat pups.

#### 4-room test (4RT)

For the 4RT, we constructed a 60-cm square acrylic box partitioned into four rooms using black opaque acrylic boards. A rat pup of PND11 that was unknown to the test rats was placed in the corner of one of the four rooms, and immediately after, the test rat was placed in the center of the square box. The illuminance was 41 and 24 lx at the center of the apparatus and in the corners, respectively. The pups were only used once for the test. The rats’ behavior was recorded for 5 min by overhead video recording. Two persons who did not know the experimental conditions visually measured the time spent by the pups and the test rat in the same compartment. The measurements of the two persons were then averaged.

#### Rescuing-restrained-rat test (RarT)

A transparent acrylic rectangular box (28 × 7 × 6 cm), with doors on the front and back was constructed (Fig. 5p; Fig. S8). The doors could only be opened by pushing from the outside, not from the inside. The door was marked with vertical black lines so that the rats could easily recognize it. A male rat of the same age as the test rats was restrained in the box, which was fixed on the floor of the OF arena where the comatose rat in TT were fixed, and the same area (40 × 25 cm) as the live zone in TT was designated as the Rescue zone. Then, a test rat was released from one of the corners of the OF arena, and the movement was recorded for 5 or 10 min using the video-tracking system and a video camera. A restrained rat was used for two tests involving the EAH and CNT rats. The duration spent in the Rescue zone and the time of first entry into the zone were measured using the video-tracking system. Illuminance was the same as that in the OFT.

#### Third-party punishment test (TPPT)

An observation box (40 × 30 × 30 cm) for TPPT was constructed using acrylic boards (Fig. 2). The rear and right sides were made with translucent gray acrylic boards, where an experimenter could observe the animals’ behavior. An opaque black acrylic board with a rough surface was used for the bottom of the box. The other parts were made of transparent acrylic boards. At the top, we placed a transparent acrylic lid with 4 cm-diameter holes in four corners to prevent animals from escaping. Using two removable partitions, we divided the apparatus into three rooms: the ICR, BL6, and Rat rooms. The center of the apparatus was illuminated at 47 lx. The partitions had holes with a diameter of 1 cm at approximately 4.5 cm intervals, 5 cm from the bottom, so that the animals could smell each other. A cosmetic brush that was attached to a 1 cm-square acrylic rod (40 cm long) was used to stop any dangerous attacks between the animals that may cause injuries (see Fig. S4). Video cameras were set up on the front and left sides of the box to record the animal behaviors (Fig. 2). The floor of the box was covered with a 2 cm-thick floor bedding. The test was conducted using a 11- to 13-week-old male ICR mouse and a male BL6 mouse younger by 1 week than the ICR mouse in addition to the actor rat. First, an aggressive pair of ICR and BL6 mice were placed in the box without partitions to confirm that the ICR mouse attacked the BL6 mouse within 1 min. Then, the mice were removed, and an actor rat was put into the box, allowing them to move freely for 2 min to acclimate to the environment as well as the brush, which was moved slowly in front of the rat. Next, we inserted a central partition to separate the rat and mouse rooms and then placed the aggressive pair in the mouse room side so that the rats could observe the aggression by the ICR mouse for 3 min. If the ICR mouse attacks did not reach 5 times, the time was extended until five attacks occurred. Thereafter, we separated the mice by inserting a partition between the ICR and BL6 rooms. The rat was left in this condition for 2 min to calm itself down from agitation caused by watching the attack. Duration staying in each mouse zone by rats and the frequency sniffing each mouse were measured during this 2 min as described in Fig. 4. Finally, we removed all the partitions to allow the three animals to contact each other, and observed them for 3 min. During this time, violent contacts between the animals may occur, but could be stopped using the brush (Fig. S4). In the experiments with the peaceful pairs were conducted performed one day after completing those with the aggressive pairs, rat habituation was omitted. The partition between the mouse and rat rooms was then inserted to allow the rat to observe the interaction of the peaceful pair for 1 min. We measured the number of attacks by the ICR mouse on the BL6 mouse for 3 min and the number of contacts with the ICR mouse or BL6 mouse by the actor rats' forepaws. After removing the rat from the box, we recorded the behavior of the two mice for 1 min. Three persons (an undergraduate, a laboratory staff, and a secretary) who were unfamiliar with the experimental conditions counted the number of contacts by the actor rats with the ICR mice or BL6 mice and the number of ICR mouse attacks for 3 min when the three animals could contact each other. They also examined whether the ICR mouse attacked the BL6 within 1 min after the rat was removed.

### RNA sequencing (RNAseq)

RNA-seq for gene expression profiles in mPFC of CNT and EAH rats wsas done as described elsewhere^42^. Each RNA library was prepared using TruSeq RNA sample prep kit v2-setA (Illumina, CA, USA) according to the manufacturer’s instructions and subjected to RNA-seq on a MiSeq NGS sequencer (Illumina).

### Quantitative RT-PCR (qPCR)

qPCR was done and the results were processed as described elsewhere using the following primers ^43^.

### *Rattus* norvegicus Fos proto-oncogene, AP-1 transcription factor subunit (Fos), mRNA

Fwd sequence: AGCCGACTCCTTCTCCAGCA

Rvs sequence: AAGTTGGCACTAGAGACGGACAGAT

NCBI Reference Sequence: NM_022197.2

### *Rattus* norvegicus ribosomal protein S20 (Rps20), mRNA

Fwd sequence: TGCGGTGAAGGTTCCAAGAC

Rvs sequence: AACCTCCACTCCAGGCTCAA

NCBI Reference Sequence: NM_001007603.4

### *Rattus* norvegicus oxytocin/neurophysin I prepropeptide (Oxt), mRNA

Fwd sequence: GCATCTGCTGTAGCCCGGATGG

Rvs sequence: ATGGGGAATGAAGGAAGCGCC

NCBI Reference Sequence: NM_012996.3

Rattus norvegicus glyceraldehyde-3-phosphate dehydrogenase (Gapdh), mRNA.

Fwd sequence: GAGACAGCCGCATCTTCTTG

Rvs sequence: TGACTGTGCCGTTGAACTTG

NCBI Reference Sequence: NM_017008.4

### Immunofluorescence histochemistry

After euthanization by 100% carbon dioxide inhalation, CNT and EAH rats were subjected to perfusion fixation using a previously described procedure^44^. The PFC was coronally cryo-sectioned at a thickness of 10 µm and subjected to immunohistochemical staining with a mixture of a rabbit polyclonal antibody to Rps20 [CSB-PA040274; 2BScientific (Kidlington, UK)], a mouse monoclonal antibody to CD11b (OX-42, Bio-Rad, Hercules, CA, USA), and a guinea pig polyclonal antibody to NeuN (ABN90P; Merck Millipore, Burlington, MA, USA). The specimens were observed as described elsewhere^44^.

### Statistical analysis

Data are expressed as the mean ± SD. Statistical data were analyzed using two-tailed unpaired or paired *t*-test, χ^2^ test with Fisher’s exact test, one- or two-way analysis of variance (ANOVA) with Tukey’s post hoc test, or Spearman correlation analysis (the method used in each experiment is described in the figure legends). All analyses were conducted using Prism 8 (GraphPad Software, La Jolla, CA, USA). A *p*-value less than 0.05 was considered significant for all tests. The single, double, triple, and quadruple asterisks in the graphs indicate statistical significance at *p* < 0.05, 0.01, 0.001, and 0.0001, respectively.

## Supplementary Information

Supplementary Information 1.

Supplementary Video 1.

Supplementary Video 2.

Supplementary Video 3.

Supplementary Video 4.

Supplementary Video 5.

## Data Availability

The majority data are available in the main text or the supplementary materials. Any other data will be provided upon request to JT, a corresponding author.
