# Publisher Correction: Third-party punishment-like behavior in a rat model

**DOI:** 10.1038/s41598-024-76454-2

**Published:** 2024-10-22

**Authors:** Kanta Mikami, Yuka Kigami, Tomomi Doi, Mohammed E. Choudhury, Yuki Nishikawa, Rio Takahashi, Yasuyo Wada, Honoka Kakine, Mayuu Kawase, Nanae Hiyama, Hajime Yano, Naoki Abe, Tasuku Nishihara, Junya Tanaka

**Affiliations:** 1 Department of Molecular and Cellular Physiology, Ehime University Graduate School of Medicine, Shitsukawa, Toon, Ehime 791‑0295 Japan; 2 Department of Anesthesia and Perioperative Medicine, Ehime University Graduate School of Medicine, Shitsukawa, Toon, Ehime 791‑0295 Japan

Correction to: *Scientifc Reports* 10.1038/s41598-024-71748-x, published online 27 September 2024

The original version of this Article contained an error in the Funding section.

This study is reported in accordance with ARRIVE guidelines. The animal experiments were approved by the Ethics Committee for Animal Experimentation of Ehime University (Approval No.: 05U43-2; 05U47-2; 05U46-1, 2).

now reads:

This work was supported by the Japan Society for the Promotion of Science (JSPS) Grant-in- Aid for Challenging Research (Exploratory) 20K21465 to JT; Grant-in-Aid for Scientific Research (C) to TN (19K09436; 23K08383); Grant-in-Aid for Scientific Research (C) to MEC (24K12069).

The original Article has been corrected.

