# Supplementary material for: Third-party punishment-like behavior in a rat model
Source: Sci Rep. 2024 Sep 27;14:22310. doi: 10.1038/s41598-024-71748-x (PMC11436845; doi:10.1038/s41598-024-71748-x)
Supplement: Supplementary file 1 — Supplementary Information 1. [file 41598_2024_71748_MOESM1_ESM.docx]

Supplementary Materials for

Third-party punishment using a rat model

Kanta Mikami^1^, Yuka Kigami^1^, Tomomi Doi^1^, Mohammed E. Choudhury^1^, Yuki Nishikawa^1,2^, Rio Takahashi^1^, Yasuyo Wada^1^, Honoka Kakine^1^, Mayuu Kawase^1^, Nanae Hiyama^1^, Hajime Yano^1^, Naoki Abe^2^, Toshihiro Yorozuya^2^, Tasuku Nishihara^2^, and

Junya Tanaka^1*^

^1^ Department of Molecular and Cellular Physiology, Ehime University Graduate School of Medicine, Shitsukawa, Toon, Ehime, 791-0295, Japan

^2^ Department of Anesthesia and Perioperative Medicine, Ehime University Graduate School of Medicine, Shitsukawa, Toon, Ehime, 791-0295, Japan

*Corresponding author. Email: jtanaka@m.ehime-u.ac.jp

**This includes:**

Fig. S1 to S9

**
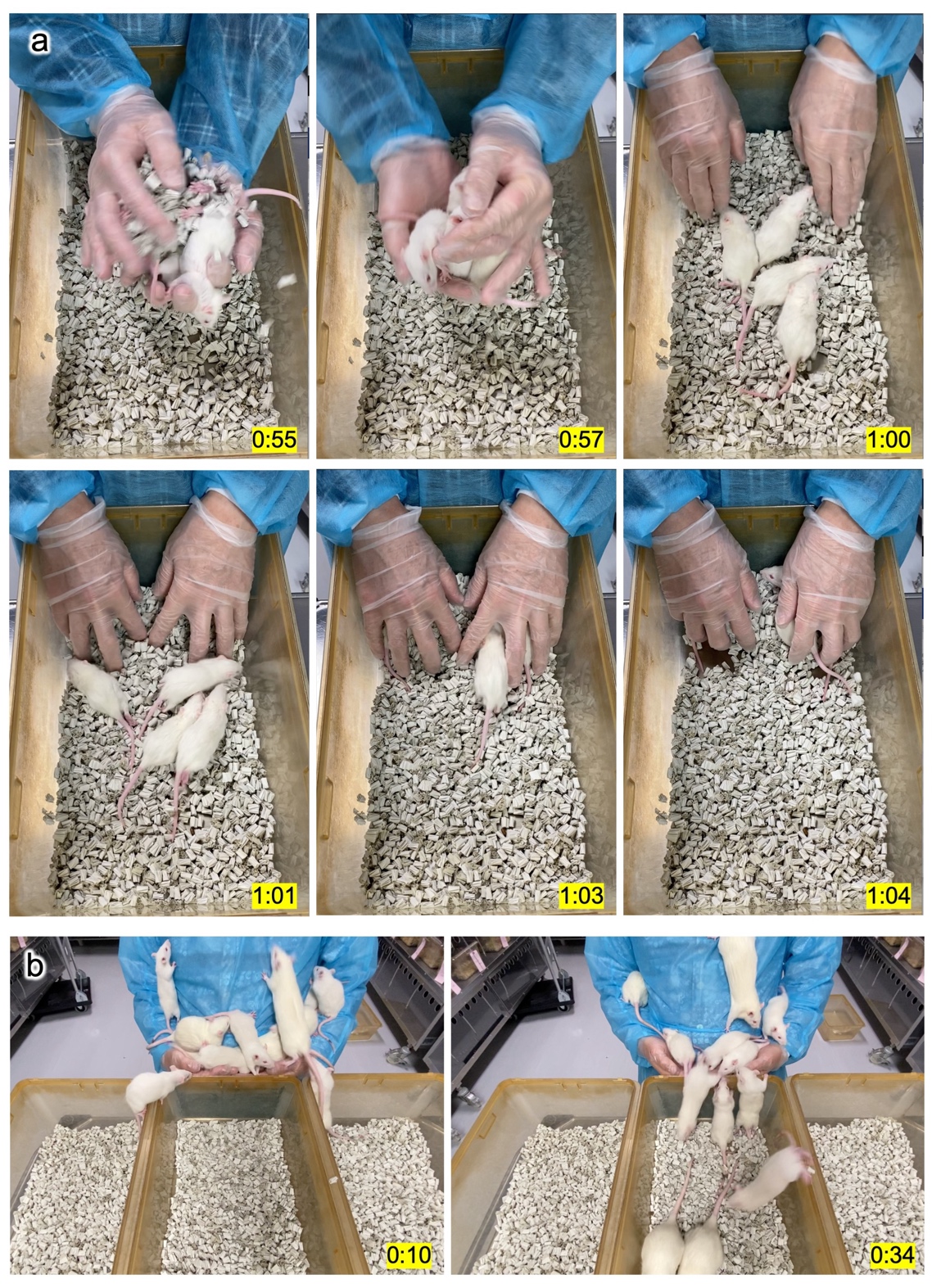
**

Fig. S1.

**EAH procedure; playing with rats.** Video-captured images. a) Playing with four PND25 rats in hands of the caretaker, spinning them around together. The rat pups appeared so happy that they gathered around to ask the caretaker to do it again and again. b) Group play with PND 30 and 8-week-old rats. Numbers in the yellow shadings denote the time (min:s) after the start of the recording.


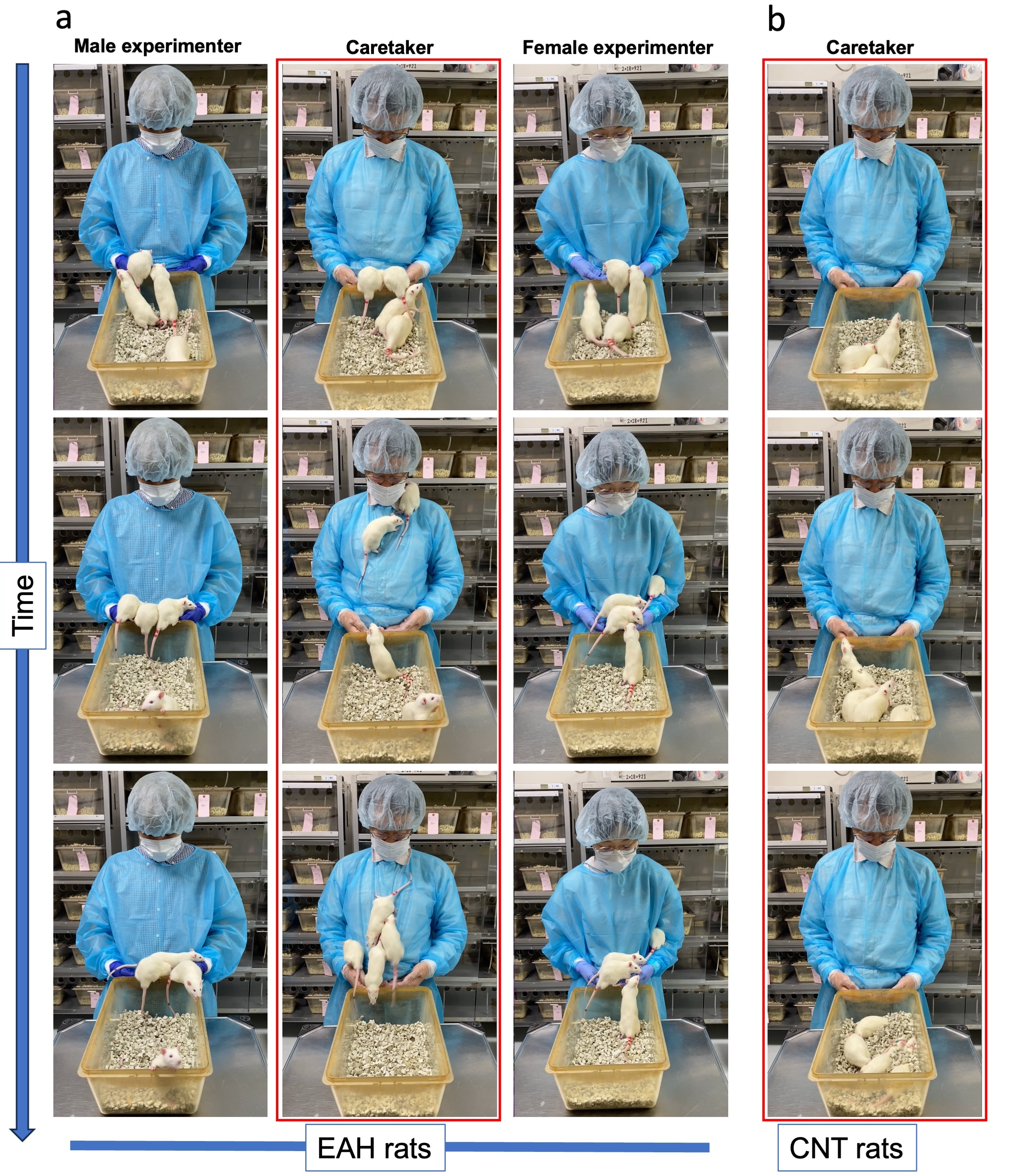


Fig. S2. Familiarity to the caretaker and unknown experimenters

The EAH group could distinguish the caretaker and unknown behavioral experimenters. a) A male experimenter in his 20s, a male caretaker in his 60s, and a female experimenter in her 20s stood with their palms outstretched in front of an EAH cage in this order, and the movements of the rats were video-recorded for 30 s. The EAH rats gathered to the palms of the unknown experimenters, while they climbed to the shoulders of the caretaker. b) CNT rats, which were litters of the EAH rats, never approached the caretaker who routinely cared for them.

**Fig. S3. A representative result in a case of an EAH rat with an aggressive mice pair.**

Video-captured images showing that an EAH rat tried to stop aggression of an ICR mouse toward a BL6 mouse. A detailed experimental procedure and a representative result are shown. The rat in this figure is different from that shown in other Figures. Numbers in the yellow shading are the time (min:s) after the start of the recording.


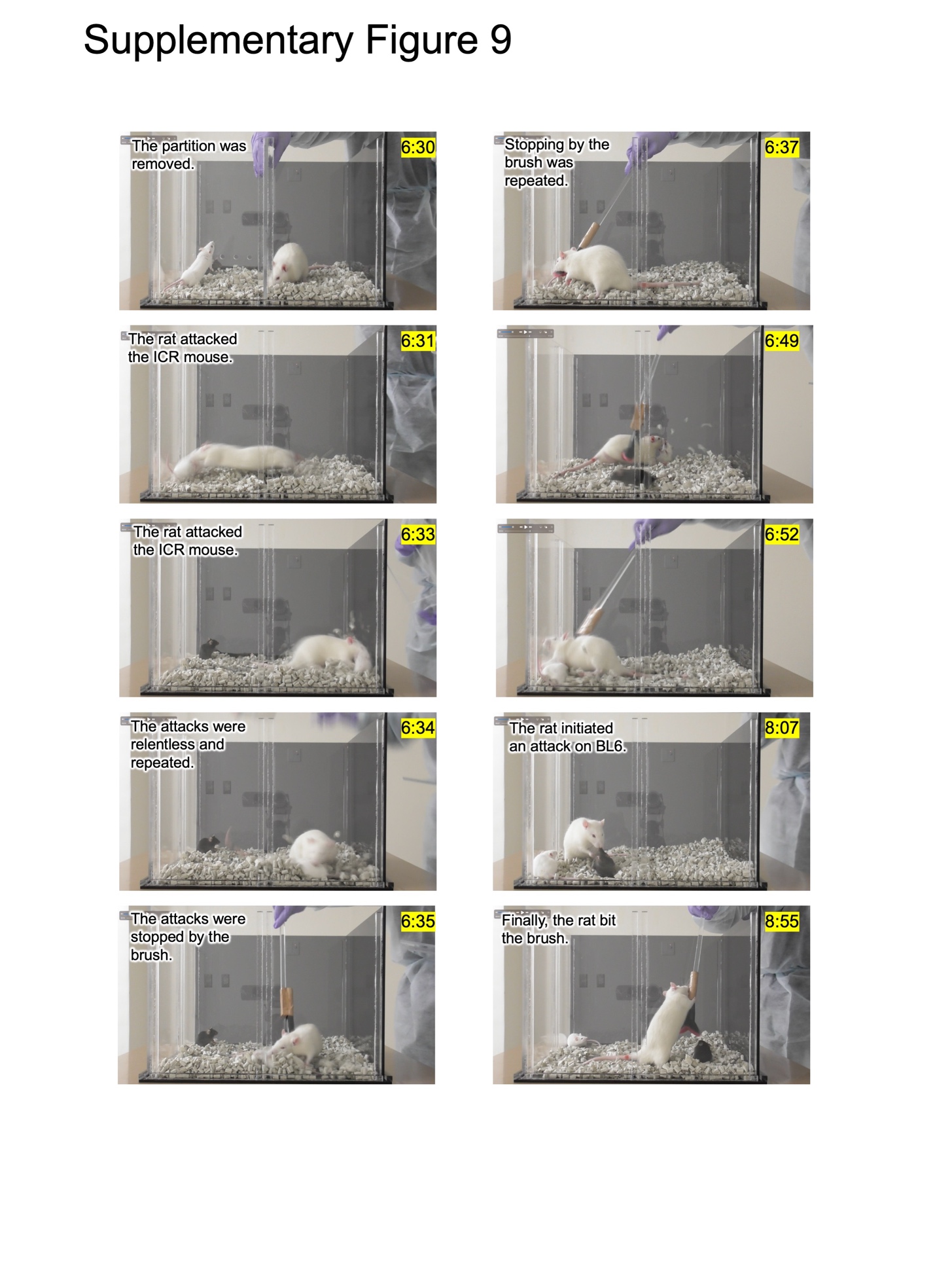


**Fig. S4. An aggressive behavior of an EAH rat**

The number of contacts with the mice by an EAH rat shown here could not be correctly counted because the rat repeatedly attacked the mice with resisting the brush. Therefore, these data were not included in the graph shown in Fig. 2E and 2H. Numbers in the yellow shading denote the time (min:s) after the recording was started. The rat contacted the aggresive ICR mouse promptly after the transparent partition was removed at 6:30. The rat contacted the ICR mouse repeatedly and aggressively. To protect the mice from the rat’s attack, the experimenter stopped them repeatedly with a brush that was attached to an acrylic rod. Probably because of the repeated stopping by the brush, the rats began to contact the BL6 mouse instead of the ICR mouse at 8:07. Finally, the rat started to bite the brush at 8:55.

**Fig. S5. Time-lapse images of the rat movements in DT**

Numbers in the yellow shadings denote the time (min:s) after the start of the recording. Rat pups at PND16 were placed in the left room (with 6 cm-deep water) at 2:28 (CNT) or 2:34 (EAH), and a transparent partition was inserted between the left and center rooms. The rats observed the drowning pup vigorously swimming in the left room for 1 min. After partition removal at 3:33 (CNT) or 3:45 (EAH), the EAH rat entered the center room (with 3 cm-deep water) and touched the pup within 30 s, whereas the CNT rat never entered the center room. The EAH rat seemingly tried to help the pup enter the right room, but the attempts seemed unsuccessful. The CNT rat did not help the pup throughout the observation.

Fig. S6. Difference between behaviors of a CNT rat and an EAH rat in TT

Images are shown captured from the videos recording the movements of the rats in the live zone in TT for 5 min. The two rats stayed for approximately 120 s in the live zone. The EAH rat frequently touched the head of the comatose rat, but the CNT rat barely touched it (the same comatose rat as that for the EAH rat).

**
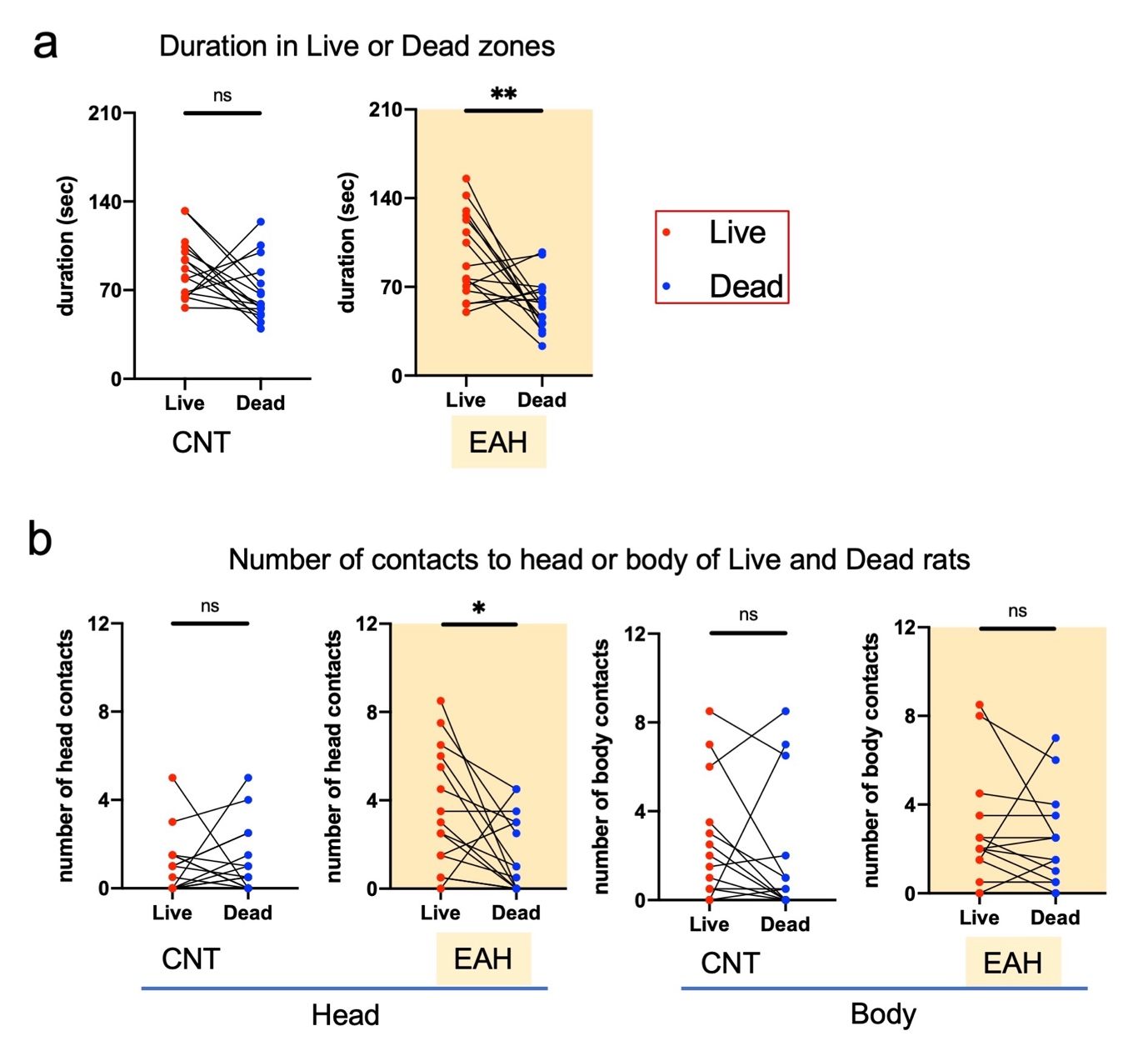
**

**Fig. S7. Movements of CNT and EAH rats in TT**

a) Comparison of the rats’ duration of stay in the live and dead zones in TT. The EAH rats stayed for longer periods in the Live zone than in the Dead zone. b) Number of contacts with the head or body of the comatose and euthanized rats in the live and dead zones, respectively. The EAH rats contacted with comatose rats’ heads more frequently than the euthanized rats’ heads. *n* = 16, Paired two-tailed *t*-test. *, ** indicate statistical significance at p < 0.05, 0.01, respectively.

**Fig. S8. Rescue of an unknown restrained rat by a CNT rat**

Video-captured images showing that a CNT rat rescued an unknown restrained rat in RarT. The numbers in the yellow shadings denote the time (min:s) after the start of the recordings. This CNT rat was the only one among 32 rats tested that could rescue the restrained rat within 6 min. The CNT rat tried to rescue the restrained rat from the back door, but finally, it succeeded in rescuing the restrained rat from the front door.

**Fig. S9 Behaviors of rats reared in five different conditions evaluated with OFT.**

a) Total moved distance, b) Frequency entering the center zone, c) Duration staying there. One-way ANOVA and Tukey’s post hoc test. *, **, ***, **** indicate statistical significance at p < 0.05, 0.01, 0.001, and 0.0001, respectively.
